# Enhanced systemic tumor suppression by in situ vaccine combining radiation and OX40 agonist with CpG therapy

**DOI:** 10.1186/s12967-023-04504-w

**Published:** 2023-09-12

**Authors:** Zhichen Sun, Yanhong Chu, Jie Xiao, Yueling Yang, Fanyan Meng, Xinyue Wang, Yanbing Dong, Junmeng Zhu, Yirong Wu, Lanqun Qin, Yaohua Ke, Baorui Liu, Qin Liu

**Affiliations:** 1 The Comprehensive Cancer Centre of Nanjing Drum Tower Hospital, The Affiliated Hospital of Nanjing University Medical School, Nanjing, 210008 China; 2 The Clinical Cancer Institute of Nanjing University, Nanjing, China; 3 Nanjing Drum Tower Hospital Clinical College of Nanjing University of Chinese Medicine, Nanjing, China; 4 The Comprehensive Cancer Centre of Nanjing Drum Tower Hospital, Clinical College of Nanjing Medical University, Nanjing, China; 5 Department of Medical Oncology, Fudan University Shanghai Cancer Center, Shanghai, China; 6 Department of Oncology, Shanghai Medical College, Fudan University, Shanghai, 200032 China

**Keywords:** TLR9 agonist, OX40 agonist, Radiation, In situ vaccine, Tumor microenvironment

## Abstract

**Background:**

In situ tumor vaccine has been gradually becoming a hot research field for its advantage of achieving personalized tumor therapy without prior antigen identification. Various in situ tumor vaccine regimens have been reported to exert considerable antitumor efficacy in preclinical and clinical studies. However, the design of in situ tumor vaccines still needs further optimization and the underlying immune mechanism also waits for deeper investigation.

**Methods:**

A novel triple in situ vaccine strategy that combining local radiation with intratumoral injection of TLR9 agonist CpG and OX40 agonist was established in this sturdy. Local and abscopal antitumor efficacy as well as survival benefit were evaluated in the bilateral tumors and pulmonary metastasis model of B16F10 melanoma. In situ vaccine-induced immune responses and immune-associated variation in tumor environment were further investigated using multiparameter flow cytometry and RNA sequencing. Base on the analysis, the RT + CpG + αOX40 triple in situ vaccine was combined with checkpoint blockade therapy to explore the potential synergistic antitumor efficacy.

**Results:**

Enhanced tumor suppression was observed with minimal toxicity in both treated and untreated abscopal tumors after receiving RT + CpG + αOX40 triple vaccine. The introduction of local radiation and OX40 agonist benefit more to the inhibition of local and abscopal lesions respectively, which might be partially attributed to the increase of effector memory T cells in the tumor microenvironment. Further analysis implied that the triple in situ vaccine did not only activate the microenvironment of treated tumors, with the upregulation of multiple immune-associated pathways, but also enhanced systemic antitumor responses, thus achieved superior systemic tumor control and survival benefit. Moreover, the triple in situ vaccine synergized with checkpoint blockade therapy, and significantly improved the therapeutic effect of anti-programmed cell death protein (PD)-1 antibody.

**Conclusion:**

This triple combining in situ vaccine induced intensive antitumor responses, mediated effective systemic tumor control and survival benefit, and displayed impressive synergistic antitumor effect with checkpoint blockade therapy. These data preliminary confirmed the efficacy, feasibility and safety of the triple combining in situ vaccine, suggesting its great application potential as both monotherapy and a part of combined immunotherapeutic regimens in clinical scenario.

**Supplementary Information:**

The online version contains supplementary material available at 10.1186/s12967-023-04504-w.

## Introduction

Encouraged by the striking breakthrough achieved by immune checkpoint blockades (ICB) and gene engineering T cells, immunotherapy has become the most rapidly developing field of modern oncology [1–3]. With the popularization of next-generation sequencing techniques, neoantigen identification and individualized cancer vaccines have emerged and gradually developed into a clinically effective strategy over the past few decades [4, 5]. Neoantigen-based vaccines contribute to generate and amplify antigen-specific T cell responses, and have demonstrated considerable therapeutic effect when used alone or combined with other treatments in both preclinical researches and early clinical trials [6–11]. However, there are still limitations restricting the widespread and universal application of existing immunotherapies. For instance, patients with tumors lacking of lymphocytes infiltration (generally referred as “cold tumors”) usually fail to benefit from therapeutic strategies such as ICB [12, 13]. While the time-consuming and laborious preparation process of neoantigen-based vaccines or gene-engineering immune cell therapies greatly hampers the extensive clinical application of these strategies.

In situ vaccine is an immunotherapeutic strategy which exploits tumors as antigen repertoire to induce tumor-specific immune response, in which process tumor itself was converted into an endogenous vaccine [14]. On the one hand, in situ vaccines are directly generated from in vivo tumor tissues, which can provide multiple neoantigens meanwhile circumvent the cumbersome manufacture of custom-made neoantigen vaccines, including whole exon sequencing (WES), neoantigen screening and synthesis. On the other hand, in situ vaccine serves as a priming therapy which helps to break the immune tolerance of tumor microenvironment. It thus would be beneficial to non-inflamed “cold” tumors and increase the response rate of some other immunotherapies [15, 16].

To improve the immunogenicity of tumor and turn it into a “self-vaccine”, various strategies have been introduced to the formulation of in situ vaccines, including chemotherapy, radiotherapy, immunostimulatory agents and so on [14]. CpG oligodeoxynucleotides (CpG ODNs), an agonist of toll-like receptor (TLR)-9, is one of the most widely studied synthetic immunostimulatory agent. The ligation of CpG may increase the production of Type I interferon (IFN), promote antigen presentation by antigen presenting cells (APCs), and induce the generation of adaptive immune responses [14, 17]. CpG has been tested in clinical trials either alone or in combination with other therapies. Even though single-agent CpG has shown evidence of antitumor activity and good tolerability in numerous early clinical studies, considering the limited effect of this monotherapy, CpG was currently mainly explored as a part of combination treatment regimens [18–21]. Given that the immunomodulatory role of radiotherapy has now been established, the combination of CpG and radiation has been evaluated in serval studies. Local irradiation induces immunogenic cell death (ICD) and antigen release of tumor cells, facilitates the priming and recruitment of immune cells, thus synergizes with CpG administration. It has been demonstrated that the combined remedy achieved considerable tumor suppression in both preclinical studies and early clinical trials [22–27]. In addition, the combination of CpG with other immunomodulatory agents has also been extensively investigated, among which the combination of CpG with OX40 agonistic antibody displayed fairly impressive efficacy. OX40 is one of the costimulatory molecules of T cell activation, belonging to the tumor-necrosis factor (TNF) receptor superfamily [28]. OX40 signaling can promote the proliferation and survival of T cells, contribute to the effector T cells function and the establish of long-term T cell memory responses [29]. It has been reported that intratumoral administration of low doses CpG and OX40 agonistic antibody triggered tumor-specific T cell response, suppressed the growth of the injected, as well as the untreated abscopal tumors and achieved significantly prolonged survival [30]. Nevertheless, these in situ vaccine regimens performed somewhat inconsistently in different studies, and the antitumor efficacy still needs further improvement.

Here, we developed a novel in situ vaccine strategy combining CpG, local radiotherapy (RT) and OX40 agonist (αOX40), and evaluated its antitumor efficacy in various melanoma bearing mouse models. Our study demonstrated that the combination of RT + CpG + αOX40 elicited a stronger systemic antitumor immune response, converted the tumor microenvironment (TME) into a more immunogenic condition, achieved superior antitumor efficacy than either previously reported dual combined regimen on local, abscopal tumor control and overall survival, meanwhile, cooperated to improve the responsiveness towards ICB therapy.

## Methods

### Reagents

CpG ODN 2359 was obtained from Tsingke Biological Technology Co. (Beijing, China). Anti-OX40 agonistic antibody (αOX40, Clone OX-86) was provided by GenScript USA Inc. Murine anti-PD-1 antibody (G4C2) was provided by Shanghai Junshi Biosciences Co.,Ltd (Suzhou, China).Monoclonal antibodies (mAbs) used for flow cytometry were listed as flow: FITC anti-mouse CD45 (Biolegend, USA), PE/Dazzle™ anti-mouse CD3 (Biolegend, USA), FITC anti-mouse CD8 (Biolegend, USA), PerCP/Cyanine5.5 anti-mouse CD4 (Biolegend, USA), FITC anti-mouse CD11c (Biolegend, USA), PE anti-mouse CD86 (Biolegend, USA), PE/Cyanine7 anti-mouse CD80 (Biolegend, USA), PE anti-mouse CD44 (Biolegend, USA), PE/Cyanine7 anti-mouse CD62L (Biolegend, USA), PerCP/Cyanine5.5 anti-mouse PD-1 (Biolegend, USA), PE/Cyanine7 anti-mouse OX40 (Biolegend, USA), FITC anti-mouse CD11b (Biolegend, USA), PerCP/Cyanine5.5 anti-mouse F4/80 (Biolegend, USA), APC anti-mouse CD206 (Biolegend, USA), Mouse Regulatory T cell staining kit (eBioscience, USA).

### Cell lines

B16F10 melanoma were purchased from the Cell Bank of Shanghai Institute of Biochemistry and Cell Biology, maintained at 37 °C with 5% CO_2_ in RPMI 1640 medium supplemented with 10% fetal bovine serum, 100 U/mL penicillin and 100 µg/mL streptomycin.

### Mice

Female C57BL/6 mice aged 6–7 weeks were purchased from the GemPharmatech (Nanjing, China) and housed at the Animal Center of Affiliated Nanjing Drum Tower Hospital of Nanjing University Medical School (Nanjing, China). All procedures were conducted in accordance with the guidelines verified and approved by the Ethics Committee of the Affiliated Nanjing Drum Tower Hospital of Nanjing University Medical School.

### Correlation analysis

TLR9 signaling pathway and corresponding downstream elements were retrieved from Kyoto Encyclopedia of Genes and Genomes (KEGG) database (https://www.genome.jp/kegg/kegg2.html). An online analysis tool (http://gepia2.cancer-pku.cn/#correlation) was used to predict the relationship of TLR9 or TLR9-responsive elements and OX40 in skin cutaneous melanoma (SKCM). P values and R values were calculated by Pearson’s correlation test as indicated. *p* < 0.05 is considered statistically significant. R > 0 indicates variables are positively correlated, vice versa, R < 0 indicates variables are negatively correlated.

### Animal experiments

The Ethics Committee of Drum Tower Hospital approved all experiments in this study. All animal procedures were carried out in compliance with guidelines set by the Animal Care Committee at Drum Tower Hospital (Nanjing, China).

To investigate the correlation between the activation of TLR9 signaling pathway and OX40 expression, 5 × 10^5^ B16F10 cells were subcutaneously inoculated on C57BL/6 mice. When the largest diameter of B16F10 melanoma reached 5–6 mm, mice were randomized to three treatment groups: control, CpG and CpG + RT group. Phosphate buffer saline (PBS) or CpG ODN 2359 (25 µg in 50 µL) were injected into the tumor nodule respectively in control or CpG group. For CpG + RT group, CpG ODN 2359 (25 µg in 50 µL) were injected intratumorally after a single dose 10 Gy irradiation. Tumors were excised 48 h after treatment, and the expression of OX40 on CD4+ T cells were analyzed by flow cytometry.

For the establishment of bilateral tumor model, 5 × 10^5^ B16F10 cells were subcutaneously inoculated on the right flank of C57BL/6 mice on day 0 (primary tumors) and 2.5 × 10^5^ B16F10 cells on the left flank on day 3 (secondary tumors). On day 8, when the volume of primary tumors reached about 50–100 mm^3^, mice were randomly assigned to five treatment groups including: control, CpG, CpG + RT, CpG + αOX40, and triple combination (CpG + RT + αOX40) group. On day 8, primary tumors were locally irradiated with a single dose of 10 Gy, with a lead shield protecting the rest of the animal. CpG (25 µg in 50 µL) with or without αOX40 (4 µg in 50 µL) were administrated by intratumoral injection 2 h later, and repeated on days 10 and 12. Animals were followed, tumor growth and body weight were recorded every other day until tumors reach 20 mm in average diameter. The tumor size was determined using a digital caliper and calculated as length × width^2^ × 0.5. One week after the last treatment, mice in each group were randomly selected and sacrificed. Tumors (primary and secondary), and spleens were collected and processed for flow cytometry analysis.

For pulmonary metastasis model, 5 × 10^5^ B16F10 cells were subcutaneously inoculated on the right flank of C57BL/6 mice on day 0, and 5 × 10^5^ B16F10 cells were injected through caudal vein on 9 days post inoculation of subcutaneous tumors, one day after the beginning of first treatment. The treatment was given in a way similar with the bilateral tumor model mentioned above. On 19 days post-inoculation of primary tumors, mice were sacrificed and tumor nodes on pulmonary were counted.

For cooperative therapy of the triple in situ vaccine and immune checkpoint blockade therapy, anti-PD-1 antibody (100 µg per mouse) was intraperitoneally injected on day 9, day 11 and day 13. Tumor growth and body weight of mice were followed, recorded and calculated as above mentioned.

### Single-cell preparations

Tumors were cut into 1–2 mm^3^ pieces, digested using 1 mg/mL Collagenase IV (Sigma, USA) and 100 U/mL DNase (Sigma, USA) for 2 h at 37 °C with gentle agitation, and filtered through a 40 μm nylon mesh. Spleens were homogenized by forcing the tissue through a 40 μm nylon mesh. Cell suspensions were pelleted at 350×*g* for 5 min at 4 °C and resuspended in red cell lysis solution (Biosharp, Beijing, China) to remove red blood cells for 5 min. Cells were stored at 4 °C until further usage.

### Flow cytometry analysis

For surface staining, single-cell suspensions were blocked for 5 min on ice and then stained with anti-mouse antibodies against CD45 (30-F11), CD3 (145-2C11), CD8 (53-6.7), CD4 (GK1.5), CD44 (IM7), CD62L (MEL-14), CD11c (N418), CD11b (M1/70), CD206 (C068C2), F4/80 (BM8), CD25 (PC61), CD80 (16-10A1), CD86 (GL-1), PD-1 (29 F.1A12), OX40 (RM134L). All antibodies were used at a dilution of 1:100, and samples were incubated with antibodies for 30 min at 4 °C in the dark. Mouse Regulatory T cell staining kit (eBioscience, USA) was used to detect Foxp3, which is expressed in nucleus. The transcription factor Foxp3 was stained with anti-mouse antibodies against FoxP3 (MF-14). Flow cytometry data was collected on a 5-color CytoFLEX cytometer (Beckman Coulter, USA) or a BD Accuri C6 (BD Bioscience, USA) and analyzed in FlowJo.

### Cytotoxicity assay of mouse splenocytes

One week after the last treatment, mice from control group and CpG + RT + αOX40 group were randomly selected and sacrificed. Spleens were collected and processed to obtain single cell suspension of splenocytes. B16F10 melanoma cells were stained with CFSE for 10 min at 37 °C in dark. Then splenocytes of mice in control group or CpG + RT + αOX40 group were incubated with CFSE labeled B16F10 melanoma cells at effector target ratio (E:T) of 5:1, 10:1, 20:1 and 30:1, at 37 °C with 5% CO_2_. After 5 h-incubation, the mixed cells were stained with PI for 15 min at 4 °C in dark, and then washed twice before flow cytometry analysis. Flow cytometry data was collected on a 5-color CytoFLEX cytometer (Beckman Coulter, USA) and analyzed in FlowJo.

### mRNA sequencing and gene expression analysis

One week after the last treatment, primary tumors of mice in control group or CpG + RT + αOX40 group were excised and quickly frozen by liquid nitrogen. The mRNA samples of control group and CpG + RT + αOX40 group were extracted using RNeasy Micro Kits (Qiagen, Hilden, Germany) following the manufacturer’s instructions, and then were used for RNA-seq (BerryGenomics, Beijing, China). DESeq2 Bioconductor package was used to perform differential expression analysis. Gene Ontology (GO) and KEGG enrichment analysis were performed using the database “The Gene Ontology Resource” and “KEGG” respectively. Gene Set Enrichment Analysis (GESA) was performed using the GSEA software.

### Statistical analysis

Data analysis was performed using SPSS and GraphPad Prism 8. The assumption of normal distribution was firstly tested for all data presented. For data fits the normal distribution, P values were calculated by two-tailed unpaired Student’s t-tests or corrected Student’s t-tests as indicated according to homogeneity of variances (corrected Student’s t-tests were applied for data that disaccord with homogeneity of variances). As for data does not comply with the normal distribution, P values were calculated by non-parametric test (Mann–Whitney U test) as indicated. Log-rank (Mantel–Cox) test was used for survival analysis. *p* < 0.05 was considered statistically significant (ns *p* > 0.05, *p < 0.05, **p < 0.01, ***p < 0.001 and ****p < 0.001).

## Results

### In situ vaccine with CpG and local irradiation upregulate OX40 expression on tumor infiltrating CD4+ T cells

**Fig. 1:**
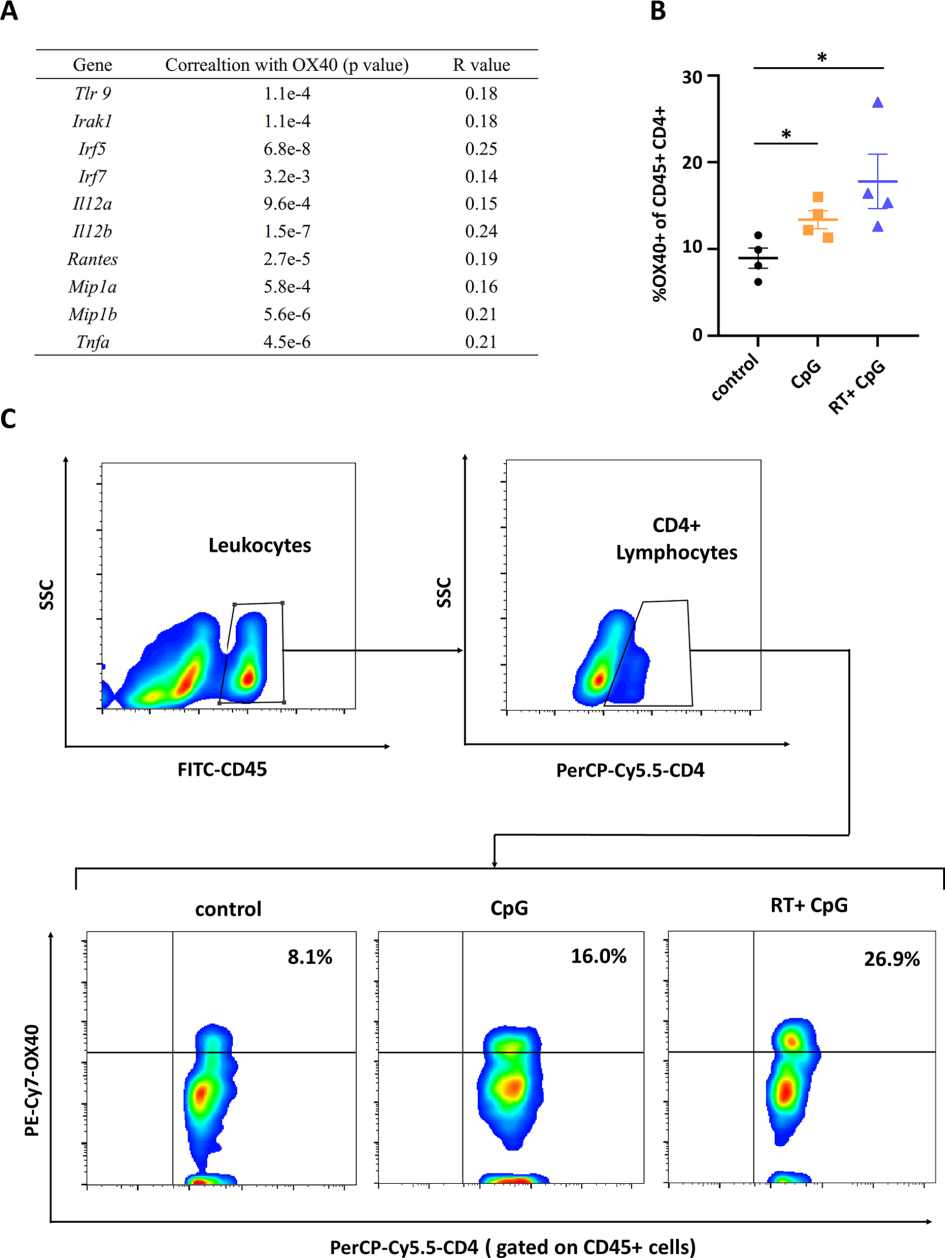
CpG and radiotherapy upregulated the expression of OX40 on tumor infiltrating CD4+ T cells in the tumor microenvironment. **A** The correlation between OX40 expression and TLR9 or TLR9 downstream elements in SKCM was analyzed by an online tool GEPIA 2. **B**, **C** 48 h after treated with PBS, CpG intratumoral injection or 10 Gy irradiation followed by CpG intratumoral injection, tumors were excised and OX40 expression on the CD45+CD4+ T cell subset was analyzed by flow cytometry (OX40+ subset gate on CD45+CD4+ cells). Data are represented as mean ± s.e.m. n = 4. Student’s t test was used for statistical analysis. *p < 0.05

It has been established in previous study that intratumoral administration of TLR9 agonists may increase OX40 expression on tumor infiltrating CD4+ T helper cells [30]. Using an online analysis tool GEPIA 2, it was demonstrated that the OX40 expression was positively correlated with TLR9 and its downstream signaling pathway in the tumor microenvironment of skin cutaneous melanoma (SKCM) (Fig. 1A). Such correlation was again verified in our study. In a B16F10 subcutaneous tumor model, OX40 expression was significantly increased, with nearly 2-fold upregulation, on infiltrating CD4+ T cells after intratumoral injection of CpG, a ligand for TLR9. In addition, there were also studies demonstrating that radiotherapy may induce OX40 expression on tumor infiltrating CD4+ T lymphocytes [31, 32]. Therefore, we investigated how the irradiation prior to CpG injection affect the OX40 expression on CD4+ T cells in tumor microenvironment. Tumors were harvested 48 h post treatment, and flow cytometry analysis demonstrated that the introduction of radiotherapy further upregulated OX40 expression on CD4+ T cells, with a significant 2.2-fold increase compared with control group, and a moderate 1.2-fold increase compared with CpG group (Fig. 1B, C). OX40 is a costimulatory molecule, and the engagement of OX40 signaling can facilitate the activation of effector memory T cells (TEM), thus promotes the antitumor immune response [28, 29, 36]. Therefore, these data suggested that local irradiation and intratumoral CpG injection further upregulate the OX40 expression on tumor infiltrating CD4+ T cells, and the triple combination of RT + CpG + αOX40 has the potential to achieve superior antitumor efficacy.

### Triple combining in situ vaccine achieved superior tumor suppression in both local and abscopal lesion, and prolongs survival with minimal toxicity

**Fig. 2:**
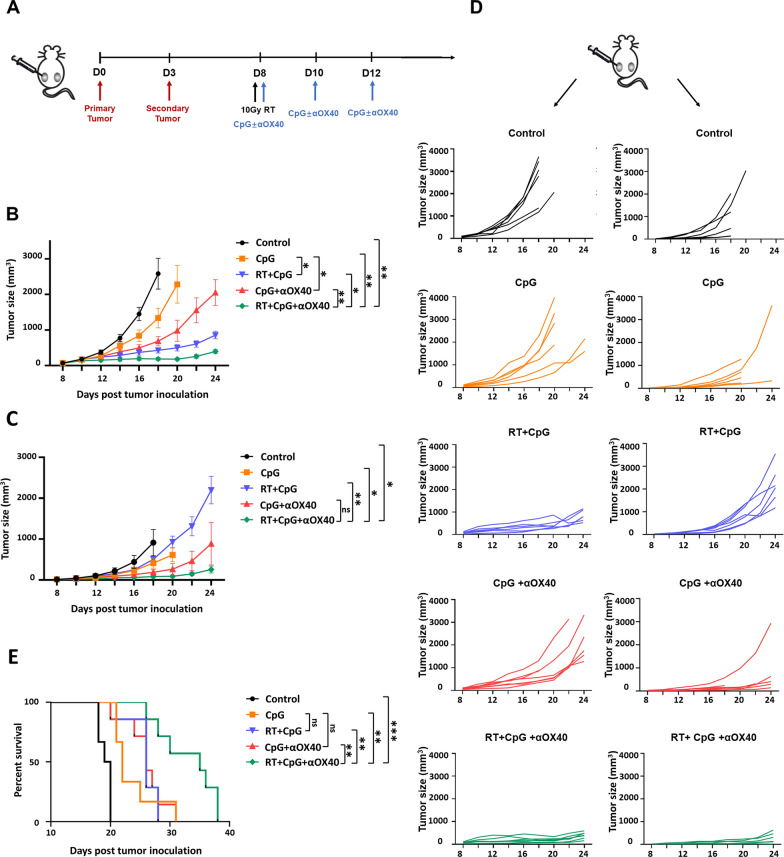
Triple combining in situ vaccine suppressed both local and abscopal tumors, and prolonged survival. **A** Treatment schema of the in situ vaccine in bilateral tumor model. C57BL/6 mice were implanted with B16F10 melanoma on the left (5 × 10^5^, on day 0) and right (2.5 × 10^5^, on day 3) lower sides of the abdomen, and received treatment on day 8, 10 and 12. **B**, **C** Growth curves represent the average volume of **B** primary (local, treated) or **C** secondary (abscopal, untreated) tumors in each group. Data are represented as mean ± s.e.m. n = 6–7. Student’s t test or corrected Student’s t test was used for statistical analysis. ns, not significant representing *p* > 0.05, *p < 0.05, **p < 0.01. **D** Tumor growth curves of each mouse in different groups (n = 6–7). **E** Survival curves of each treatment group (n = 6–9). Log-rank (Mantel–Cox) test was used for survival analysis. ns, not significant representing *p* > 0.05, *p < 0.05, **p < 0.01, ***p < 0.001

Mice-bearing two tumors derived from B16F10 melanoma were used as a model to monitor the systemic antitumor benefits of the novel triple therapeutic strategy combining radiotherapy, CpG and anti-OX40 agonistic antibody (RT + CpG + αOX40) (Fig. 2A). For primary tumors, a growth suppression of nearly 48% was achieved with monotherapy of CpG intratumoral administration compared to untreated group. The tumor suppressive effects were improved when CpG was combined with radiotherapy, as well as αOX40, and the synergistic effect was even more distinguished in the combination of CpG and radiotherapy, achieving 80% and 76% growth suppression respectively, compared to untreated and CpG monotherapy groups. Although the combination of CpG and radiotherapy caused remarkable regression of tumors at the local treated site, it failed to work equally on those distant untreated lesions. The introduction of αOX40 markedly improved the tumor suppression on distant lesions. It’s worth noting that the triple combination therapy (RT + CpG + αOX40) demonstrated superior tumor suppressive effect than any other monotherapy or binary combination groups, resulting in the most significant regression of both local and abscopal tumors (Fig. 2B–D and Additional file 1: Fig. S1A). Above results suggested the activation of an intensive, systemic antitumor immune response by the triple combination of CpG, radiotherapy and αOX40. What’s more, the triple combination treatment group also exhibited advantage on prolonging survival, with median survival time extending by 85% (vs. control group), 64% (vs. CpG group), 29% (vs. RT + CpG group) and 29% (vs. CpG + αOX40 group) respectively (Fig. 2E). Besides, the body weights of mice were monitored during the treatment and all the groups displayed similar changing patterns, suggesting considerable safety of the triple combination strategy (Additional file 1: Fig. S1B).

### Triple combining in situ vaccine protected animals from generating distant metastasis

**Fig. 3:**
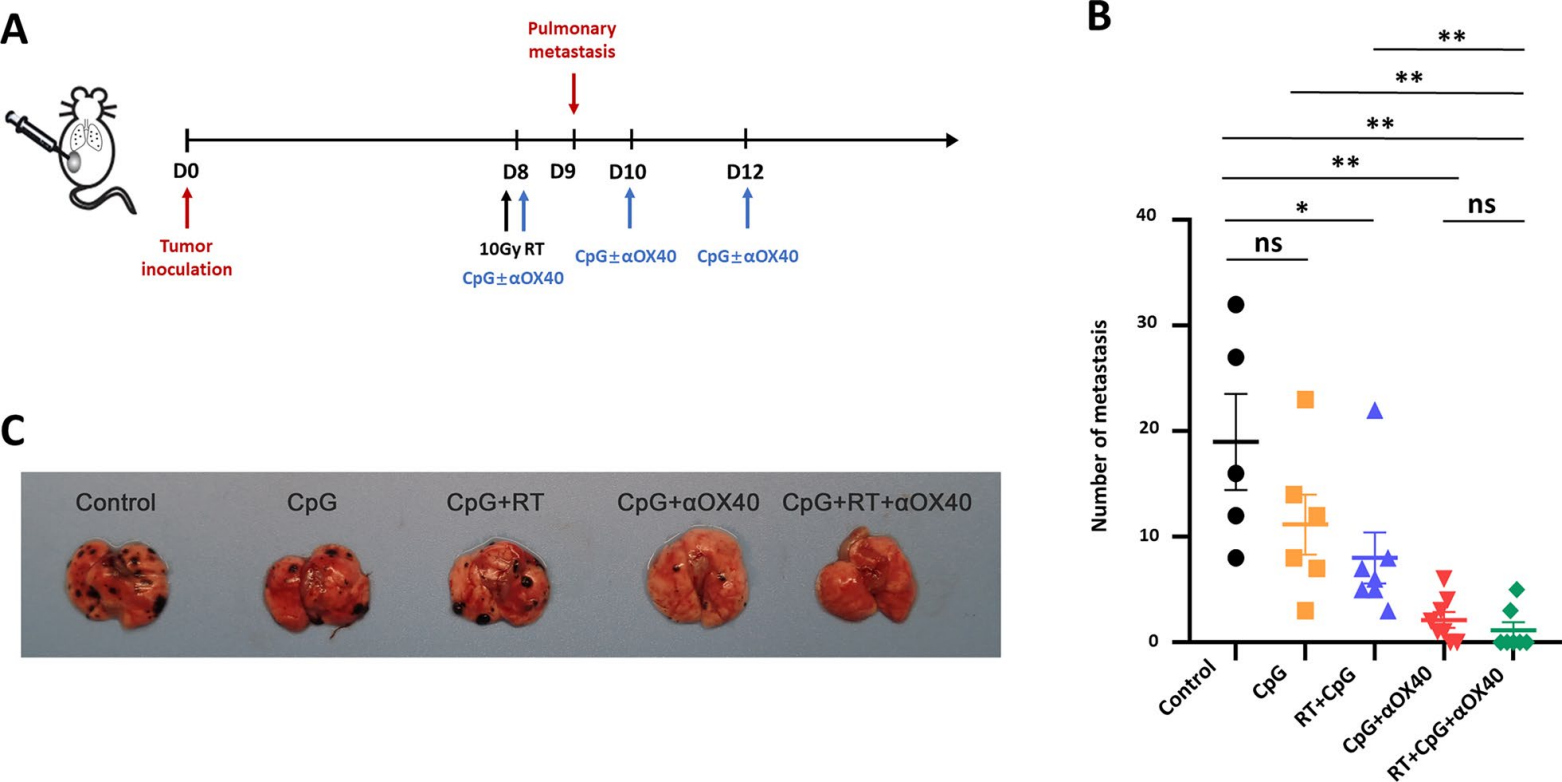
Triple combining in situ vaccine prevented the generation of distant metastasis. **A** Treatment schema of the in situ vaccine in distant metastasis model. C57BL/6 mice were implanted with B16F10 melanoma on the left lower sides of the abdomen (5 × 10^5^, on day 0), followed by injection with melanoma cells via caudal vein (5 × 10^5^, on day 9), and received treatment on day 8, 10 and 12. **B** Pulmonary metastastic foci larger than 0.5 mm in diameter were counted in different treatment groups. Data are represented as mean ± s.e.m. n = 5–8. Non-parametric test (Mann–Whitney U test) was applied for data analysis. ns, not significant representing *p* > 0.05, *p < 0.05, **p < 0.01. **C** Representative picture of pulmonary metastasis in each group

The formation of metastasis is the most mortal factor in the progression of malignant tumors. Dealing with micrometastatic foci before they are visible to medical imaging is crucial to the inhibition of tumor progression and the prolongation of life expectancy. We further investigated whether the triple combination of RT + CpG + αOX40 could prevent circulating tumor cells from forming metastasis. Mice were injected with 5 × 10^5^ B16F10 melanoma cells via caudal vein 9 days post inoculation of primary tumors to establish the model of distant metastasis, and the treatment was given as shown below (Fig. 3A). On 19 days post inoculation, mice were sacrificed and tumor nodes on pulmonary were counted. Mice in control group has 19 pulmonary metastases on average. CpG monotherapy exhibited moderate preventive effect, without statistical difference, on generation of metastasis with an average of 11 pulmonary metastastic foci. RT + CpG combination group demonstrated significant preventive effect with an average of 8 pulmonary metastastic foci.

However, CpG + αOX40 and triple combination groups demonstrated remarkable diminish of lung metastases, with an average of only 2 and 1 metastatic foci respectively (Fig. 3B, C). CpG + αOX40 and triple combination groups exhibited no significant difference in metastasis prevention, achieving 25% (2 in 8 mice) and 71% (5 in 7 mice) free of pulmonary metastasis relatively. Such result was consistent with that in bilateral tumor model, implying that the introduction of αOX40 remarkably improved the tumor suppression on untreated distant tumors. In a word, the triple combining vaccine strategy improve the control of untreated abscopal lesions, and in some degree prevent the formation of metastasis (Additional file 1: Fig. S2).

### Triple combining in situ vaccine activated the tumor microenvironment

**Fig. 4:**
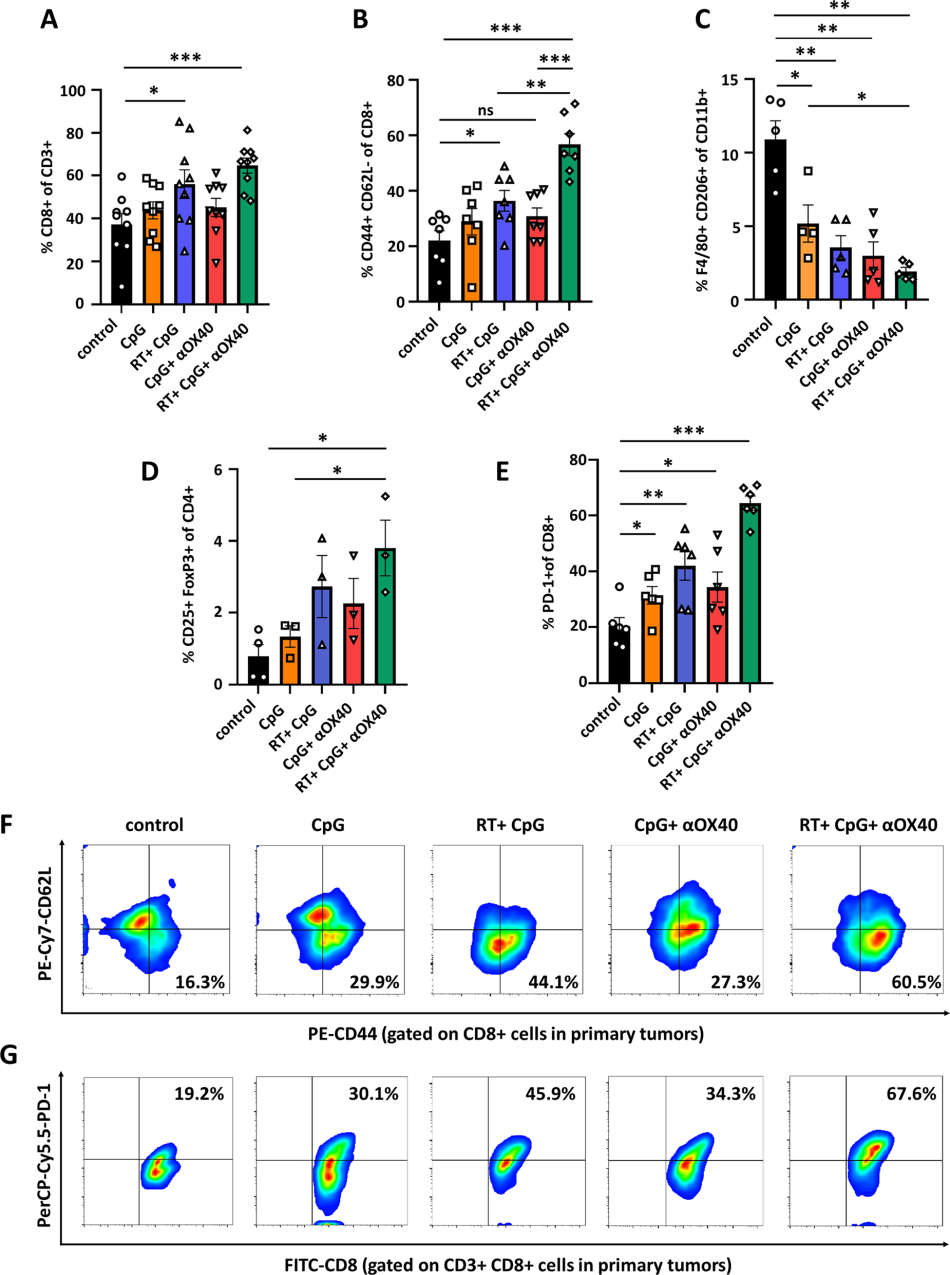
Triple combining in situ vaccine activated the tumor microenvironment. **A**–**E** Proportion of **A** cytotoxic T cells (CTLs, CD8+ gate on CD3+), **B** effector memory T cells (TEMs, CD44+CD62L– gate on CD3+CD8+ cells), **C** M2- like macrophages (M2, F4/80+CD206+ gate on CD11b+ cells), **D** regulatory T cells (Tregs, CD25+FoxP3+ gate on CD3+CD4+ cells), and **E** PD-1+ CTLs (PD-1+CD8+ gate on CD3+ cells) in in situ treated tumors of C57BL/6 mice in each group a week after the last administration. Data are represented as mean ± s.e.m. n = 3–9. Student’s t test or corrected Student’s t test was used for statistical analysis. ns, not significant representing *p* > 0.05, *p < 0.05, **p < 0.01, ***p < 0.001. **F**, **G** Representative flow cytometry images of **F** TEMs and **G** PD-1+ CTLs in in situ treated tumors of C57BL/6 mice in different groups a week after the last administration

To investigate the immune associated changes in the tumor microenvironment induced by the in situ vaccine, primary tumors were collected to analyze the immune responses by flow cytometry one week after the last treatment. Tumor-infiltrating T lymphocytes (TILs) play a crucial role in antitumor immunity. Although there was no difference in the proportion of T cells between each group, the proportion of cytotoxic T cells (CD8+ T cells) significantly increased in RT + CpG group and triple therapy group (Fig. 4A and Additional file 1: Figs. S3A, S4A). Effector memory T cells (CD3+CD8+CD44+CD62L–) were proved to induce effective immune protection and play an important role in killing tumor cells [33]. The proportions of TEMs increased significantly in RT + CpG group (36.4%), while increased moderately without statistic difference in CpG + αOX40 group (30.7%) compared to untreated group (22.1%). The highest proportion of TEMs was observed in RT + CpG + αOX40 group (56.7%), which showed marked upregulation in comparison with both doublet therapies (Fig. 4B, F and Additional file 1: Figs. S3A). As for other immune cells, although there was no difference in the M1-like macrophages among all groups, in situ vaccine reduced M2-like macrophages, and the most remarkable reduction was observed in triple therapy group, with the proportion of M2-like macrophages decreasing by more than 80% (10.9% vs. 1.9%) (Fig. 4C and Additional file 1: Figs. S3B, S4B). Consistently, the ratio of M1- to M2-like macrophages also increased by nearly 4 times in comparison to control group (0.62 vs. 2.44) (Additional file 1: Fig. S4C). Above results indicated that RT + CpG + αOX40 triple combining in situ vaccine shaped a more immunogenic tumor microenvironment, making tumors more susceptible to immune cell killing. Meanwhile, the triple in situ vaccine upregulated the regulatory T cells (Tregs) subgroup (CD3+CD4+CD25+Foxp3+), and the highest proportion of Tregs was also observed in RT + CpG + αOX40 group, with over 4-fold increase compared to untreated tumors (3.8% vs. 0.8%) (Fig. 4D and Additional file 1: Fig. S3C). Moreover, as one of the most important coinhibitory molecules, the expression of PD-1 on CD8+ TILs was also detected. The local expression of PD-1 on CD8+ TILs increased greatly after receiving triple combining in situ vaccine, reaching 3.2-fold of control group, 2.1-fold of CpG monotherapy group, 1.5-fold of RT + CpG group, and 1.9-fold of CpG + αOX40 group respectively (Fig. 4E, G and Additional file 1: Figs. S3A). The upregulation of Tregs and PD-1 expression again verified that the combining vaccine effectively induced antigen release and triggered subsequent antitumor T cell response, indicating that the triple in situ combining vaccine may further synergy with anti-PD-1 antibody or Tregs inhibitors to improve therapeutic effect [34].

### Systemic immune response induced by triple combining in situ vaccine

**Fig. 5:**
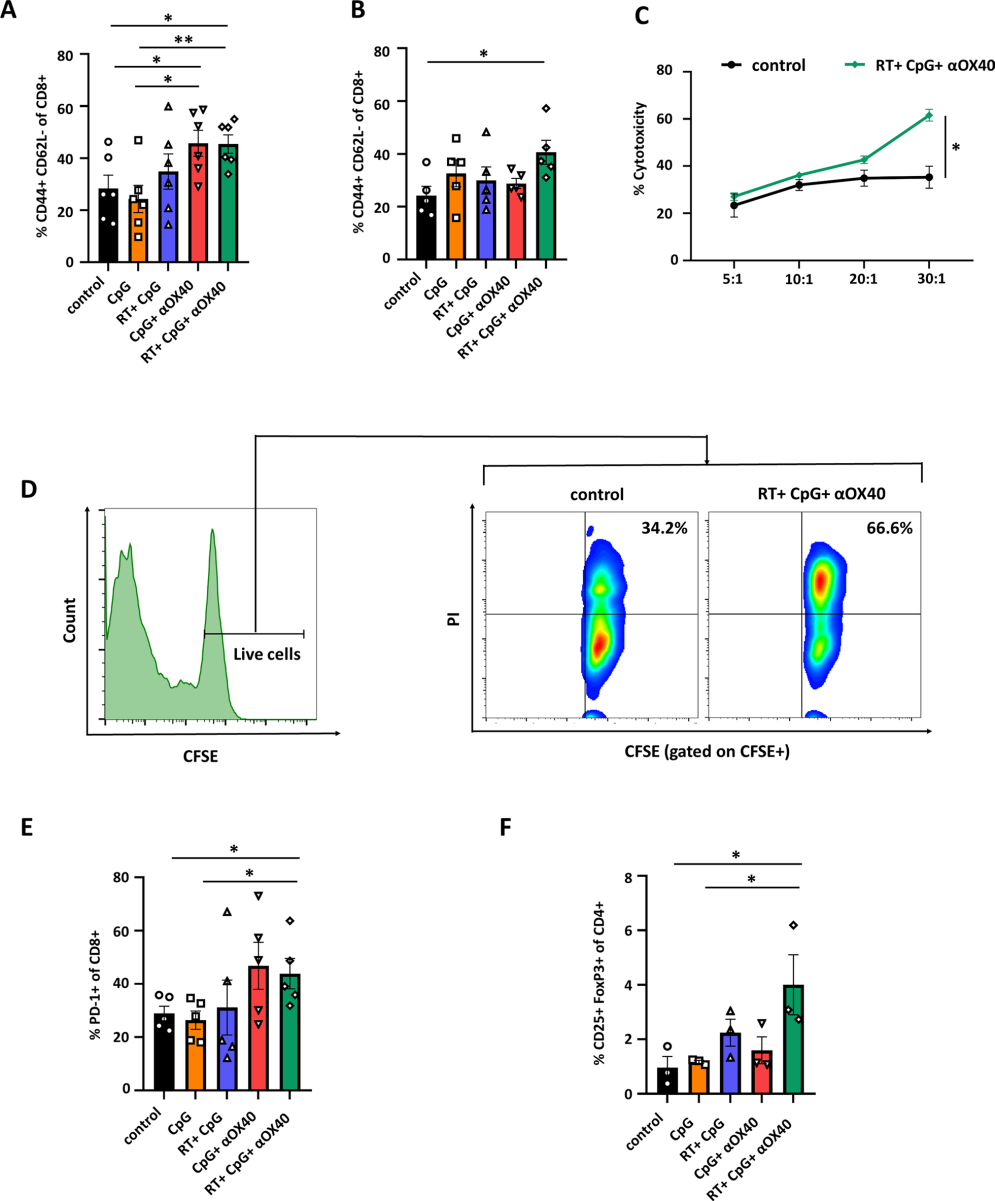
Systemic immune response induced by triple combining in situ vaccine. **A**, **B** Proportion of effector memory T cells (TEMs, CD44+CD62L– gate on CD3+CD8+ cells) in **A** abscopal tumors and **B** spleens of C57BL/6 mice in each group a week after the last administration. Data are represented as mean ± s.e.m. n = 5–6. Student’s t test was used for statistical analysis. *p < 0.05, **p < 0.01. **C** Spleen cells of mice in control group and RT + CpG + αOX40 group were incubated with CFSE labeled (indicating live cells) B16F10 melanoma cells at effector-to-target ratio (E:T) of 5:1, 10:1, 20:1 and 30:1. PI was added after 5 h-incubation and the percentage of dead cells was analyzed by flow cytometry. Data are represented as mean ± s.e.m. n = 4. Student’s t test was used for statistical analysis. *p < 0.05. **D** Representative flow cytometry plots indicating proportions of dead cells of B16F10 melanoma cells (CFSE + PI + gate on CFSE + cells). **E**, **F** Proportion of **E** PD-1+ CTLs (CD8+PD-1+ gate on CD3+ cells) and **F** regulatory T cells (Tregs, CD25+FoxP3+ gate on CD3+CD4+ cells) in abscopal tumors of C57BL/6 mice in each group a week after the last administration. Data are represented as mean ± s.e.m. n = 3–5. Student’s t test or corrected Student’s t test was used for statistical analysis. *p < 0.05

As it has been established by previous study, we observed that in situ vaccine also suppressed the growth of distant untreated tumors, which was called “abscopal effect”, suggesting the activation of systemic antitumor immune response [35]. To explore the antitumor mechanism of the in situ vaccines, secondary tumors and spleens of mice in each group were excised to evaluate the systemic immune responses. Although there was no difference in the proportion of T lymphocytes or CD8+ T cells in abscopal tumors between each group, the proportion of TEMs was significantly upregulated in CpG + αOX40 and RT + CpG + αOX40 groups. In comparison with CpG monotherapy group (24.3%), the proportion of TEMs increased moderately without statistic difference when combined with radiotherapy (34.9%), while TEMs were significantly upregulated in CpG + αOX40 (45.7%) and RT + CpG + αOX40 (45.4%) groups (Fig. 5A and Additional file 1: Fig. S5). This result was consistent with the growth curve of untreated abscopal tumors, and may partially explain the phenomenon that the introduction of anti-OX40 agonistic antibody markedly improved the suppressive efficacy towards distant lesion. Moreover, as primary and secondary tumors, increase in the proportion of TEMs was also observed in spleen cells of RT + CpG + αOX40 group (Fig. 5B). Meanwhile, the spleen cells in the triple therapy group exhibited stronger cytotoxic activity against B16F10 melanoma cells than that in control group (Fig. 5C, D). This result agreed to our aforementioned conclusion that RT + CpG + αOX40 in situ vaccine effectively prevent the formation of pulmonary metastasis, indicating the triple combining vaccine induced robust systemic tumor-specific immune response. In addition, the proportion of Tregs subgroup and the expression of PD-1 on CD8+ TILs in untreated secondary tumors were also upregulated in RT + CpG + αOX40 group compared to both control and CpG monotherapy groups, indicating the probable synergy between the triple therapy and PD-1 or Tregs blockade might also be observed in abscopal tumors (Fig. 5E, F).

### Transcriptome analysis of tumors receiving triple in situ vaccine

**Fig. 6:**
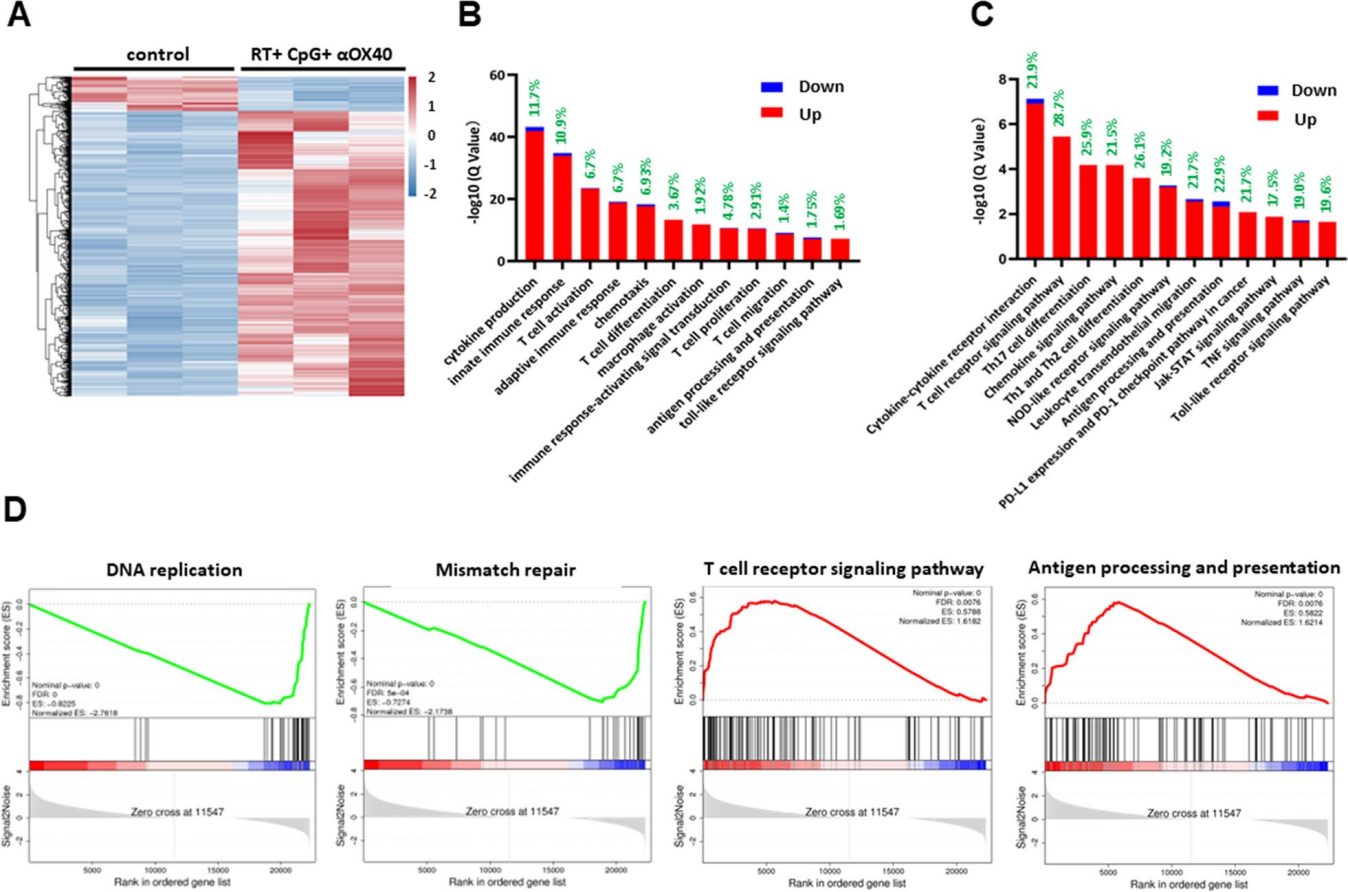
RNA-seq analysis of control tumors and tumors receiving in situ triple vaccine. **A** Heat map revealing in situ vaccine-induced changes in the gene-expression profile of the tumor microenvironment. n = 3 for both control and RT + CpG + αOX40 group. Color bars indicate normalized expression, the color from blue to red indicates that the gene expression from low to high. **B** GO enrichment analysis of up- and downregulated genes. Gene ratio (shown in green) and − log10(Q value) of all GO terms are shown. **C** KEGG enrichment analysis of up- and downregulated genes. Gene ratio (shown in green) and − log10(Q value) of all KEGG signaling pathway are shown. **D** Representative GSEA plot showing down-regulated and up-regulated enriched gene sets of all detected genes in tumors receiving in situ triple vaccine

To further investigate the immune associated changes in the tumor microenvironment comprehensively after the triple combining therapy, RNA sequencing (RNA-seq)-based transcriptome analysis was performed (Fig. 6A). RNA-seq analysis revealed that the triple vaccine regimen induced local immune stimulation, as indicated by the upregulation of genes involved in the activation of immune responses. Gene ontology (GO) analysis demonstrated that the triple vaccine amplified the cytokine production and chemotaxis, upregulated the TLR signaling pathway, promoted antigen process and presentation, T cell activation, proliferation and migration, and finally triggered innate as well as adaptive antitumor immune responses in tumor microenvironment (Fig. 6B). KEGG enrichment analysis implied more about signaling pathways. Numerous differentially expressing (mainly upregulated) genes were involved in various immune-associated signaling pathways, such as TLR signaling pathway, NOD-like receptor (NLR) signaling pathway, TNF signaling pathway, Jak-STAT signaling pathway, PD-L1/PD-1 checkpoint pathway and so on (Fig. 6C). Traditional enrichment analysis generally based on the subset of significantly differentially expressing genes, thus the individual gene with minor differential expression tend to be neglected. Gene Set Enrichment Analysis (GSEA) compensates for the lack of effective information mining of non-significantly differentially expressing genes, and explains the regulation of the functional unit (GO term or signaling pathway) more comprehensively. In GSEA-KEGG analysis, upregulation of some immune-associated signaling pathways were also observed, which consisted with results of KEGG enrichment analysis and flow cytometry analysis, again verifying a comprehensive activation of antitumor immune responses in tumor microenvironment by the RT + CpG + αOX40 vaccine (Fig. 6D and Additional file 1: Fig. S6A). Besides, GSEA-KEGG analysis also demonstrated the downregulation of DNA replication and metabolism of multiple amino acids, suggesting an overall proliferation inhibition in tumor lesions (Fig. 6D and Additional file 1: Fig. S6B).

### Combination of triple vaccine with immune checkpoint blockade therapy

The upregulation of PD-1 expression on CD8+ TILs has been observed in tumors of triple combining treated mice, moreover, transcriptome analysis demonstrated that DNA repair related pathways, such as mismatch repair, nucleotide excision repair were downregulated after receiving triple vaccine. These results preliminarily provide mechanistic basis for the sensitizing effect of the triple vaccine towards PD-1 blockade therapy (Additional file 1: Fig. S6C) [36]. Therefore, the synergistic effect of the triple therapy combined with anti-PD-1 antibody (αPD-1) was further investigated in a simple melanoma-bearing mouse model.

**Fig. 7:**
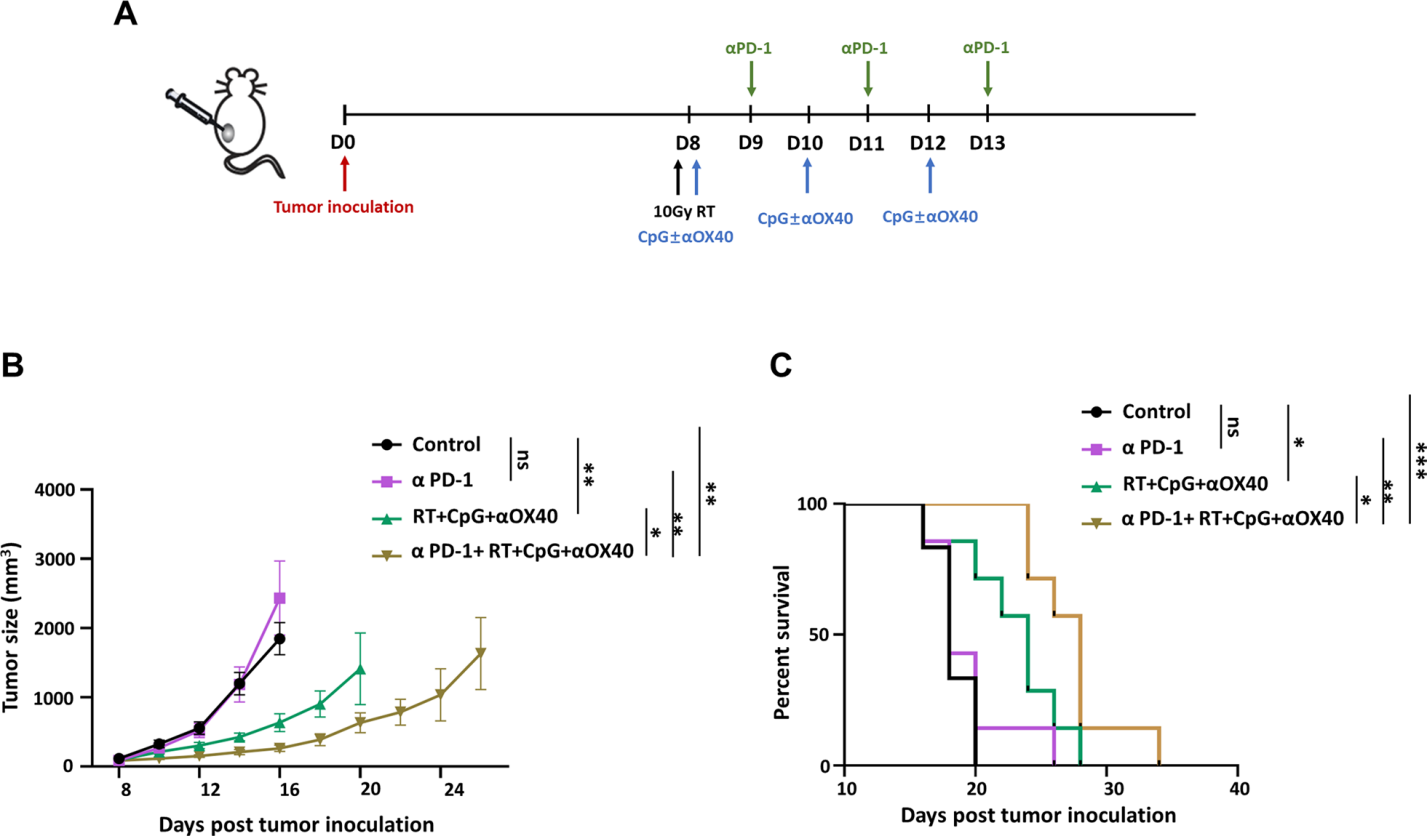
Combination of triple vaccine with immune checkpoint blockade therapy. **A** Treatment schema of the combined treatment in simple melanoma-bearing model. C57BL/6 mice were implanted with B16F10 melanoma on the left lower sides of the abdomen (5 × 10^5^, on day 0), then received in situ vaccine on day 8, 10 and 12, and αPD-1 on day 9, 11 and 13. **B** Growth curves represent the average volume of tumor in each group. Data are represented as mean ± s.e.m. n = 6–7. Student’s t test or corrected Student’s t test was used for statistical analysis. ns, not significant representing *p* > 0.05, *p < 0.05, **p < 0.01. **C** Survival curves of each mouse in different groups (n = 6). Log-rank (Mantel–Cox) test was used for survival analysis. ns, not significant representing *p* > 0.05, *p < 0.05, **p < 0.01, ***p < 0.001

Mice were randomly divided into control group, αPD-1 group, triple therapy group and triple therapy + αPD-1 group, and the treatment scheme is shown as follow (Fig. 7A). αPD-1 monotherapy exhibited no significant benefit on the suppression of tumor growth or the extension of survival time compared with untreated group, which is consistent with the consensus that non-immunogenic “cold” tumors respond poorly to checkpoint blockade therapy [37]. Nevertheless, when combining with RT + CpG + αOX40 triple vaccine, αPD-1 produced encouraging results. Remarkable antitumor effect has been observed in this cooperative strategy, achieving 89.2%, 85.8% and 58.5% growth suppression compared with untreated, αPD-1 and triple therapy group respectively (Fig. 7B and Additional file 1: Fig. S7). Moreover, the synergy of RT + CpG + αOX40 vaccine with αPD-1 demonstrated advantage on prolonging survival, with median survival time extending 10 days longer than that of control group (28 vs. 18, p = 0.0008), 9 days longer than that of αPD-1 group (28 vs. 19, p = 0.0039), and 4 days longer than of triple group (28 vs. 24, p = 0.0282) (Fig. 7C).

## Discussion

In this study, we developed an in situ vaccine combining radiotherapy, CpG and OX40 agonistic antibody to induce systemic tumor-specific immune responses that exert suppressive effect to both local (treated) and abscopal (untreated) tumors. Low dose CpG and αOX40 were injected intratumorally following local radiation, in which setting, tumor itself was converted into a “self vaccine”, exposing multiple tumor antigens for immune recognition. Therefore, this strategy achieved truly individualized tumor vaccines without requiring the pre-identification of tumor antigens and laborious preparation process, meanwhile minimized potential risk of immune escape due to limited preselected antigens [38].

The combination of CpG and OX40 has been reported to effectively suppress established tumors in several tumor models. However, the antitumor effect of this regimen in some immunologically “cold” tumors, such as 4T1 breast tumor model, remains contradictory in different studies [30, 32]. Considering that in situ vaccine strategy takes advantage of the pre-existing T cell immune repertoire within the tumor microenvironment, and possibly relies on adequate immune infiltrates to work, radiotherapy was introduced in our formulation. The immune sensitization effect of radiotherapy has been gradually realized in recent years. Radiation induces ICD characterized by the release of tumor antigens and damage-associated molecular patterns (DAMPs), leading to the upregulation of some cytokines and chemokines, which together favor immune cell priming, infiltration, and eventually systemic activation of antitumor immunity [39]. Therefore, local irradiation can sometimes elicit regression of distant unirradiated tumor as well, which was called abscopal effect [40–42]. As intratumoral administration of CpG has been reported in previous study to increase OX40 expression on CD4+ lymphocytes in TME, meanwhile, there were also studies demonstrating that radiotherapy may induce OX40 expression on tumor infiltrating CD4+ T cells [30–32]. Our data suggested that the introduction of radiotherapy further upregulate OX40 expression of CD4+ T cells on the basis of single CpG administration, providing mechanistic rationale for the triple combination of local irradiation, CpG and αOX40. We chose non-immunogenic tumor cell line B16F10 melanoma to establish tumor model. Considering B16F10 melanoma is a cell line derived from a male mouse, potential sex-related immune effects cannot be simply excluded when inoculated on female mice. Therefore, an experiment, in which male and female C57BL/6 mice with same age, kept under the same condition, were inoculated with the same amount of B16F10 cells, was conducted. Little difference was observed between tumor growth curves of different genders, preliminarily suggesting that the possible impact of sex-related immune effects on general tumor growth is negligible (Additional file 1: Fig. S8).

In bilateral B16F10 melanoma-bearing mouse model, tumor suppression was observed at both injected and distant sites after RT + CpG or CpG + αOX40 treatment, which to a certain extent reproduced the results of previous studies with similar in situ vaccine regimens. As expected, RT + CpG + αOX40 vaccine further enhanced the antitumor effect in both treated and untreated abscopal tumors in comparison with RT + CpG and CpG + αOX40 regimens, performing better in both tumor growth suppression and survival prolongation. Moreover, our results implied that radiotherapy contributes more to the control of treated lesion, while the intratumoral injection of αOX40 contributes more to the control of untreated abscopal lesion. Flow cytometry analysis partially explained the phenomenon. CTLs are generally regarded as the major executor of antitumor immune response, among which TEMs secret pro-inflammatory cytokines, such as TNF-α and IFN-γ, mediating immediate effector function, thus play an important role in tumor inhibition and killing [43, 44]. At treated tumors, infiltration of CTLs significantly increased in RT + CpG and RT + CpG + αOX40 groups, meanwhile, TEMs in tumor microenvironment were also upregulated remarkably in those two groups. While for untreated abscopal tumors, significant upregulation of TEMs was observed in CpG + αOX40 and RT + CpG + αOX40 groups.

Besides effector memory T cells, other immune cells were also investigated in tumor microenvironment. Tumor associated macrophages were polarized from M2-like phenotype into M1-like phenotype, indicating the triple vaccine changed the tumor microenvironment from immunosuppressive to immunostimulatory state [45, 46]. However, Tregs and coinhibitory molecular PD-1 were also upregulated after receiving triple vaccine. Although OX40 receptor engagement has been demonstrated to suppress the differentiation and activity of Tregs, there are also researches suggesting that the action of tumor vaccines was usually companied by a subsequent upregulation of Tregs and PD-1 expressing CD8+ T cells in tumor microenvironment [47–50]. Considering the complicated effect of radiotherapy on tumor immunity, it is explicable that the triple vaccine exerted considerable antitumor efficacy, meanwhile induced the upregulation of immunosuppressive Tregs and PD-1 expression. Further optimizing the timing and scheduling of the triple combined vaccine might contribute to a better performance in reversing immunosuppressive microenvironment and overall antitumor efficacy, and provide insights into optimal clinical translation. What is noteworthy is that these results indicated the synergistic potential of RT + CpG + αOX40 triple vaccine with PD-1 or Tregs blockade. Following combined therapy confirmed the hypothesis. In the non-immunogenic B16F10 melanoma model, systemic PD-1 antibody monotherapy failed to suppress tumor progression, while RT + CpG + αOX40 triple vaccine reactivated the therapeutic potential of checkpoint blockade, and synergize with αPD-1 to further augment tumor suppression and prolong survival.

This study developed a practical in situ vaccine strategy combining local irradiation and intratumor injection of low dose CpG and OX40 agonist, which can achieve individualized therapy without requiring custom-made and prior knowledge of the tumor antigens. This triple vaccine strategy activated tumor microenvironment, driving it from non-immunogenic to immunogenic, induced and amplified antitumor T cell responses throughout the body against tumors at treated and nontreated sites. The efficacy of inhibiting tumor growth and extending survival have been verified in melanoma-bearing models. What’s more, the triple in situ vaccine remarkably improved the therapeutic effect of PD-1 antibody monotherapy, potentially broadening its application.

## Conclusion

In summary, this study established a novel triple combining in situ vaccine strategy with favorable efficacy, feasibility and safety, and preliminary confirmed its great potential for clinical translation singly or as a part of combined immunotherapeutic regimens.

### Supplementary Information

**Additional file 1: Figure S1.** Antitumor effect and safety assessment of the triple combining in situ vaccine. (A) Individual tumor growth curves until the day of death of each mouse in different groups. (B) Weight records of mice in every treatment group, no obvious weight loss was observed. Data are represented as mean ± s.e.m., n = 6–7. Student’s t test was used for statistical analysis. ns, not significant representing *p* > 0.05. **Figure S2.** Representative picture of pulmonary metastasis of individual mouse in each treatment group. **Figure S3.** Gating strategies for all flow cytometric analyses. (A) Gating strategies for Figs. 4A, B, E and 5A, B, E. (B) Gating strategies for Fig. 4C. (C) Gating strategies for Figs. 4D and 5F. **Figure S4.** The influence of triple combiningin situ vaccine on the tumor microenvironment of treated tumors. (A) Proportion of T cells (CD3+ gate on CD45+), (B) M1-like macrophages (M1, F4/80+CD86+ gate on CD11b+), and (C) M1/M2 proportion in treated tumors of C57BL/6 mice in each group a week after the last administration. Data are represented as mean ± s.e.m. n = 4–5. Student’s t test was used for statistical analysis. ns, not significant representing *p* > 0.05, *p < 0.05, **p < 0.01. **Figure S5.** The influence of triple combining in situ vaccine on the tumor microenvironment of untreated abscopal tumors. (A, B) Proportion of (A) T cells (CD3+ gate on CD45+) and (B) cytotoxic T cells (CTLs, CD8+ gate on CD3+) in untreated abscopal tumors of C57BL/6 mice in each group a week after the last administration. Data are represented as mean ± s.e.m. n = 4–9. Student’s t test was used for statistical analysis. ns, not significant representing *p* > 0.05.**Figure S6.** GSEA plot of some notable up-regulated and down-regulated enriched gene sets in tumors receiving triple in situ vaccine. (A) Up-regulated enriched gene sets associated with immune activation. (B, C) Down-regulated enriched gene sets associated with (B) amino acids metabolism and (C) DNA repair. **Figure S7.** Individual tumor growth curves of each mouse in different groups (n = 6–7). **Figure S8.** Tumor growth curves of different genders. Male and female C57BL/6 mice aged 6–7 weeks, kept under the same condition, were subcutaneously inoculated with 5 × 10^5^ B16F10 cells. Tumor growth curves were recorded as mentioned in “Methods”. Data are represented as mean ± s.e.m., n = 5. Student’s t test was used for statistical analysis. ns, not significant representing *p* > 0.05.

## Data Availability

The datasets used and/or analyzed during the current study are available from the corresponding author (liuqin@nju.edu.cn) upon reasonable request.

## Abbreviations

PD-1: Programmed cell death protein-1
ICB: Immune checkpoint blockades
WES: Whole exon sequencing
CpG ODNs: CpG oligodeoxynucleotides
TLR: Toll-like receptor
IFN: Interferon
APCs: Antigen presenting cells
ICD: Immunogenic cell death
TNF: Tumor-necrosis factor
RT: Radiotherapy
αOX40: OX40 agonist
TME: Tumor microenvironment
PBS: Phosphate buffer saline
SKCM: Skin cutaneous melanoma
TILs: Tumor-infiltrating T lymphocytes
CTLs: Cytotoxic T cells
TEMs: Effector memory T cells
Tregs: Regulatory T cells
RNA-seq: RNA sequencing
GO: Gene ontology
NLR: NOD-like receptor
GSEA: Gene Set Enrichment Analysis
αPD-1: Anti-PD-1 antibody
DAMP: Damage-associated molecular patterns
