# Supplementary material for: Enhanced systemic tumor suppression by in situ vaccine combining radiation and OX40 agonist with CpG therapy
Source: J Transl Med. 2023 Sep 12;21:619. doi: 10.1186/s12967-023-04504-w (PMC10498626; doi:10.1186/s12967-023-04504-w)
Supplement: Supplementary file 1 — Additional file 1: Figure S1. Antitumor effect and safety assessment of the triple combining in situ vaccine. (A) Individual tumor growth curves until the day of death of each mouse in different groups. (B) Weight records of mice in every treatment group, no obvious weight loss was observed. Data are represented as mean ± s.e.m., n = 6–7. Student’s t test was used for statistical analysis. ns, not significant representing p > 0.05. Figure S2. Representative picture of pulmonary metastasis of individual mouse in each treatment group. Figure S3. Gating strategies for all flow cytometric analyses. (A) Gating strategies for Figs. 4A, B, E and 5A, B, E. (B) Gating strategies for Fig. 4C. (C) Gating strategies for Figs. 4D and 5F. Figure S4. The influence of triple combiningin situ vaccine on the tumor microenvironment of treated tumors. (A) Proportion of T cells (CD3+ gate on CD45+), (B) M1-like macrophages (M1, F4/80+CD86+ gate on CD11b+), and (C) M1/M2 proportion in treated tumors of C57BL/6 mice in each group a week after the last administration. Data are represented as mean ± s.e.m. n = 4–5. Student’s t test was used for statistical analysis. ns, not significant representing p > 0.05, *p < 0.05, **p < 0.01. Figure S5. The influence of triple combining in situ vaccine on the tumor microenvironment of untreated abscopal tumors. (A, B) Proportion of (A) T cells (CD3+ gate on CD45+) and (B) cytotoxic T cells (CTLs, CD8+ gate on CD3+) in untreated abscopal tumors of C57BL/6 mice in each group a week after the last administration. Data are represented as mean ± s.e.m. n = 4–9. Student’s t test was used for statistical analysis. ns, not significant representing p > 0.05.Figure S6. GSEA plot of some notable up-regulated and down-regulated enriched gene sets in tumors receiving triple in situ vaccine. (A) Up-regulated enriched gene sets associated with immune activation. (B, C) Down-regulated enriched gene sets associated with (B) amino acids metabolism and (C) DNA repair. Fi [file 12967_2023_4504_MOESM1_ESM.docx]

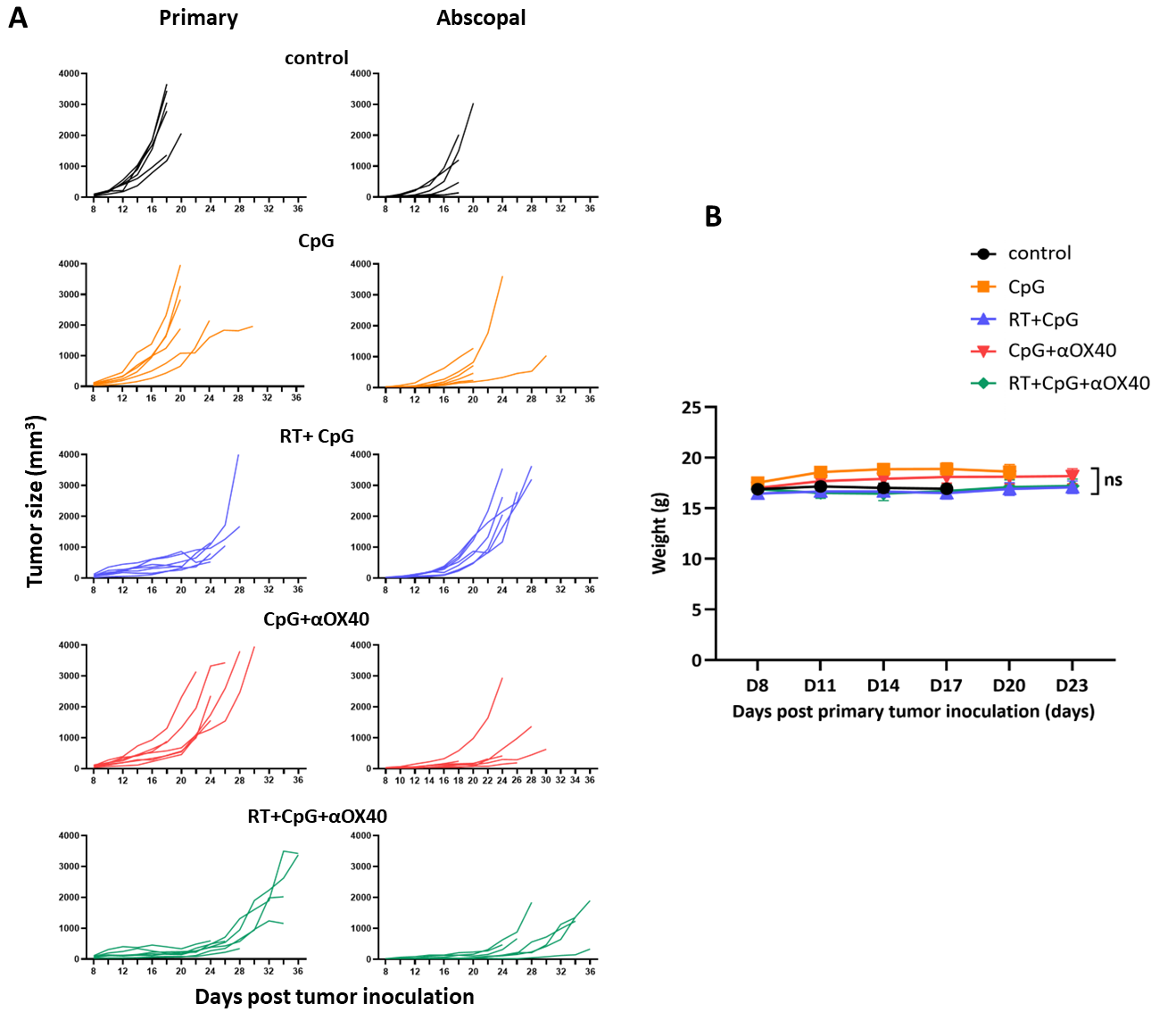


**Figure S1. Antitumor effect and safety assessment of the triple combining *in situ* vaccine.** (A) Individual tumor growth curves until the day of death of each mouse in different groups. (B) Weight records of mice in every treatment group, no obvious weight loss was observed. Data are represented as mean ± s.e.m., n=6-7. Student’s t test was used for statistical analysis. ns, not significant representing *p* > 0.05.


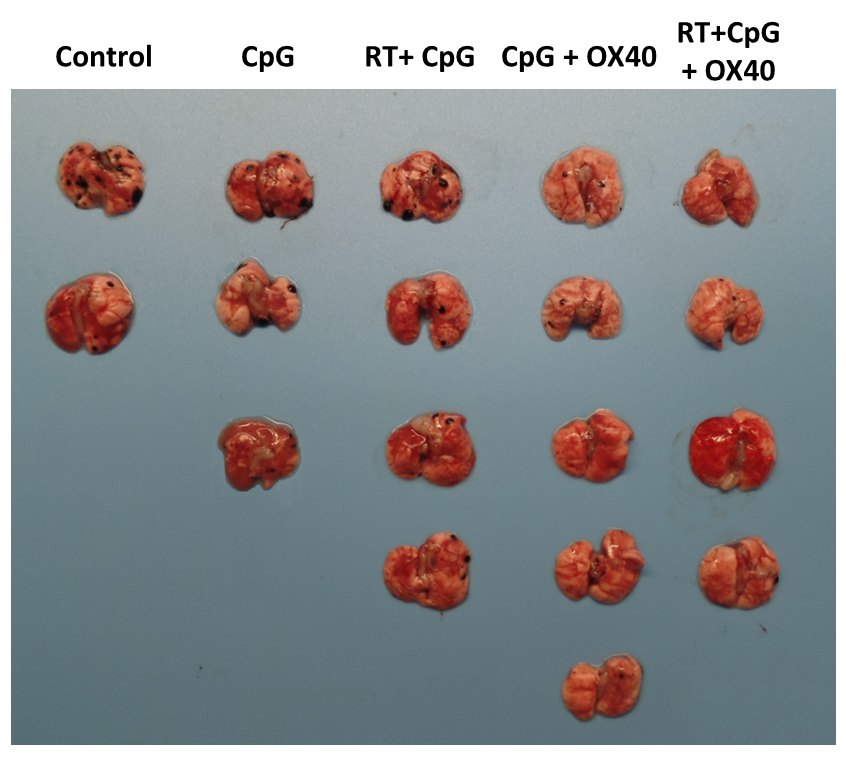


**Figure S2. Representative picture of pulmonary metastasis of individual mouse in each treatment group.**

**
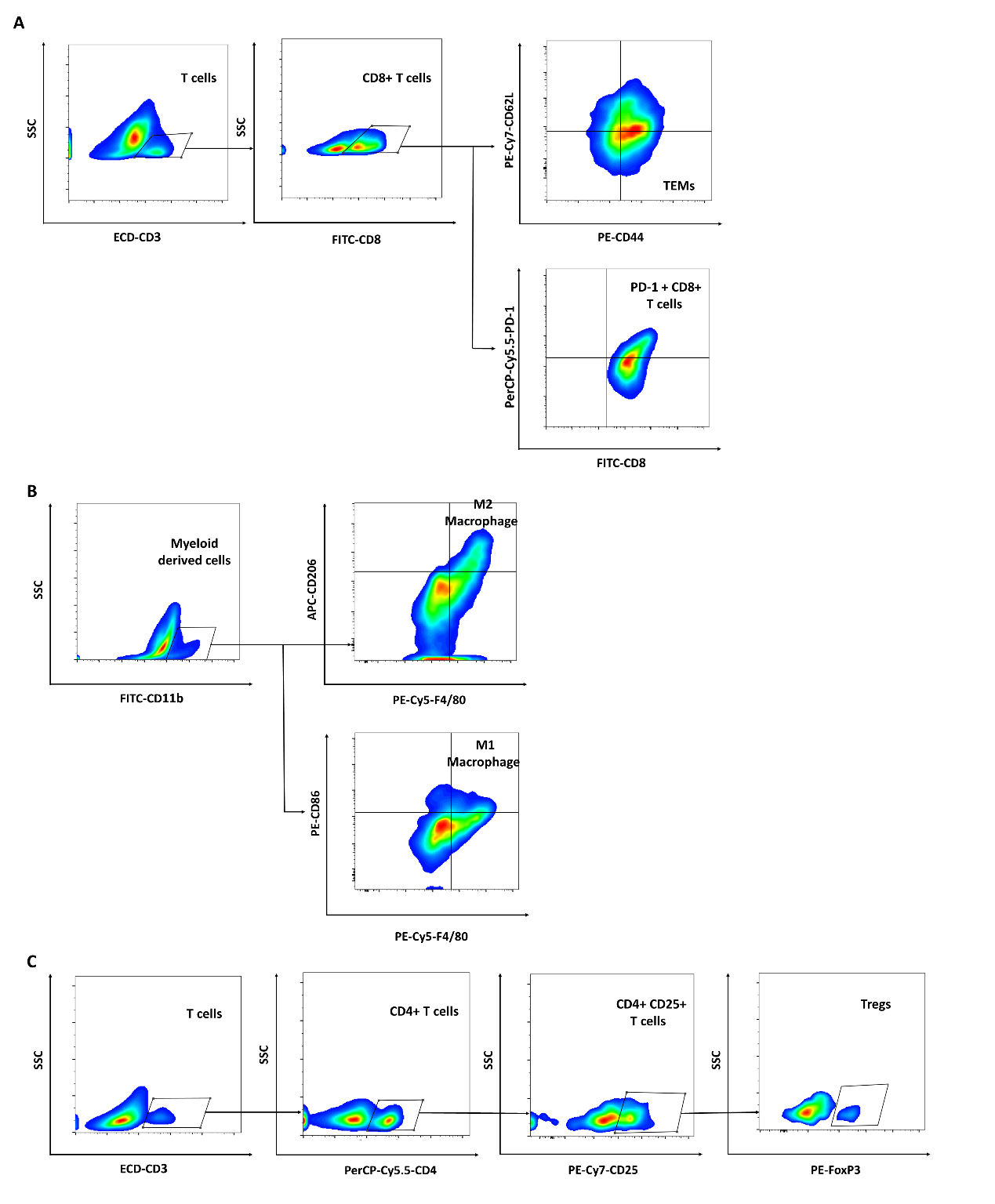
**

**Figure S3. Gating strategies for all flow cytometric analyses.** (A) Gating strategies for Figure 4A, B, E and Figure 5 A, B, E. (B) Gating strategies for Figure 4C. (C) Gating strategies for Figure 4D and Figure 5F.

**
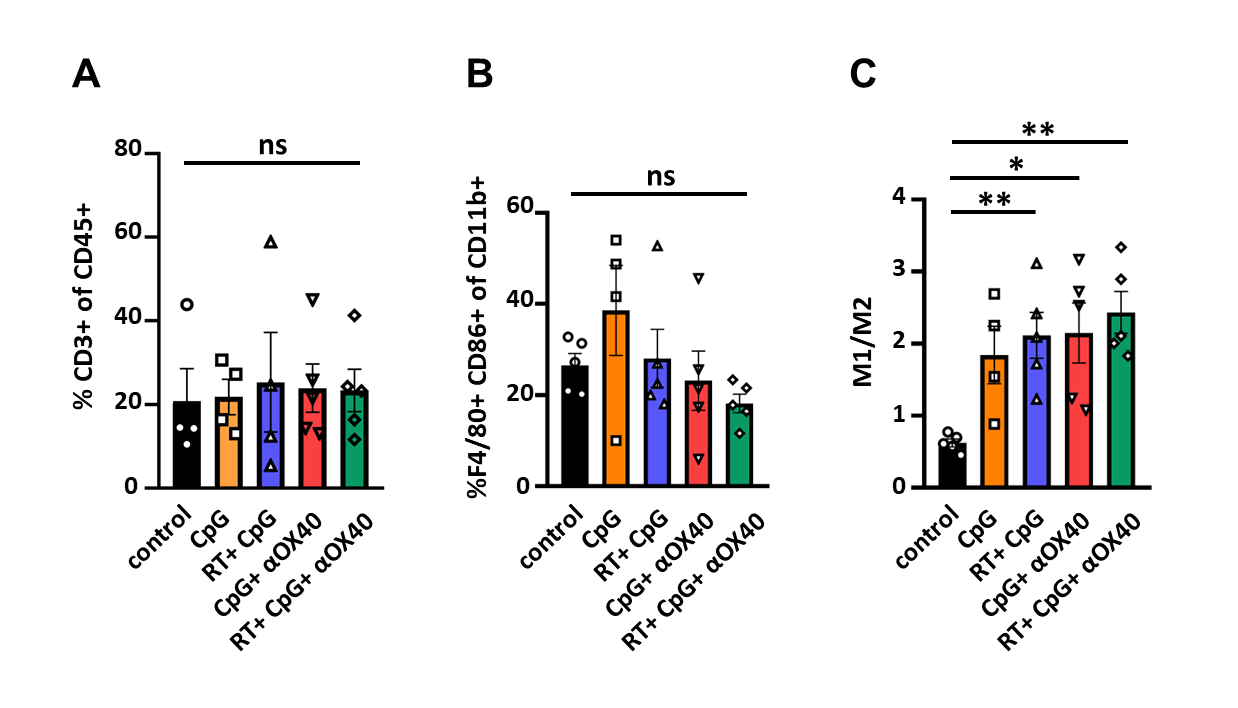
**

**Figure S4. The influence of triple combining *in situ* vaccine on the tumor microenvironment of treated tumors.** (A) Proportion of T cells (CD3+ gate on CD45+), (B) M1-like macrophages (M1, F4/80+CD86+ gate on CD11b+), and (C) M1/M2 proportion in treated tumors of C57BL/6 mice in each group a week after the last administration. Data are represented as mean ± s.e.m. n=4-5. Student’s t test was used for statistical analysis. ns, not significant representing *p* > 0.05, **p* < 0.05, ** *p* < 0.01.


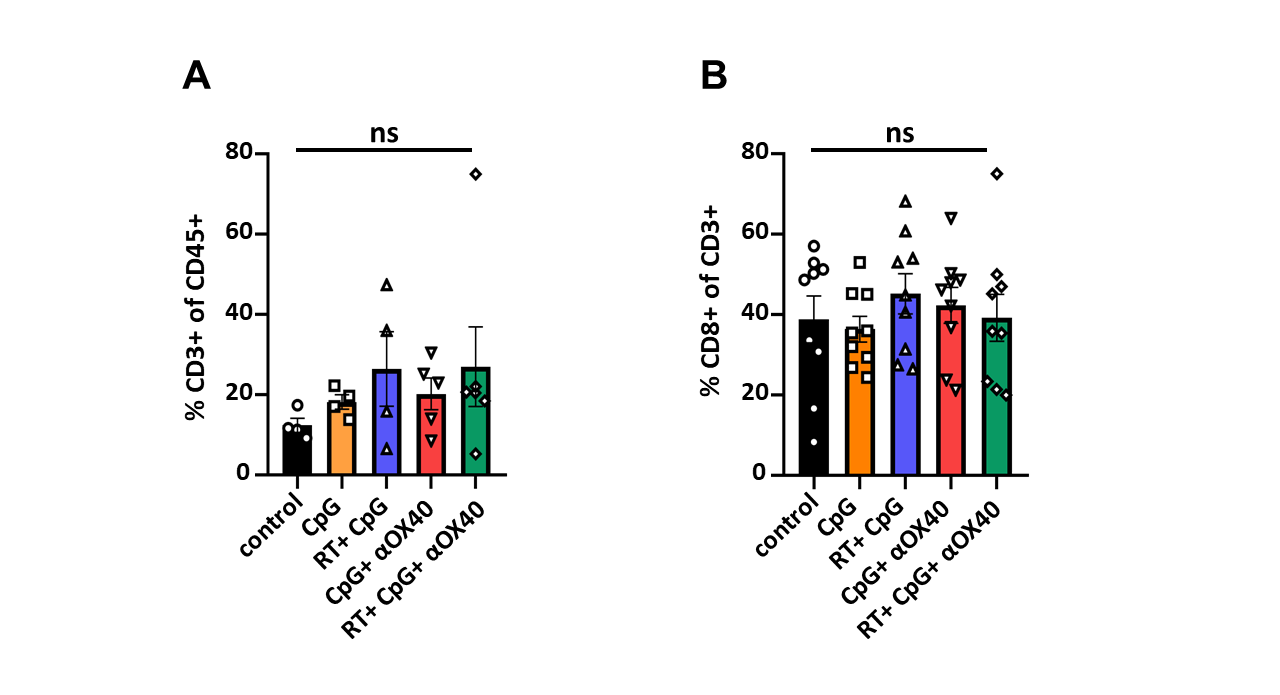


**Figure S5. The influence of triple combining *in situ* vaccine on the tumor microenvironment of untreated abscopal tumors.** (A, B) Proportion of (A) T cells (CD3+ gate on CD45+) and (B) cytotoxic T cells (CTLs, CD8+ gate on CD3+) in untreated abscopal tumors of C57BL/6 mice in each group a week after the last administration. Data are represented as mean ± s.e.m. n=4-9. Student’s t test was used for statistical analysis. ns, not significant representing *p* > 0.05.


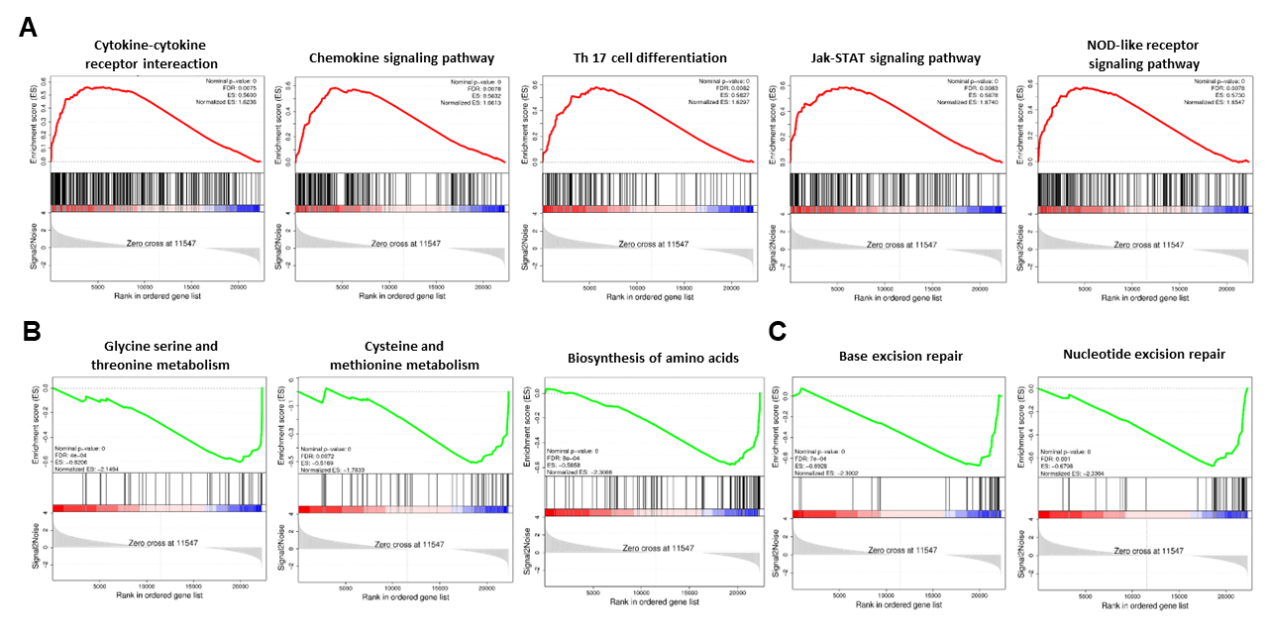


**Figure S6. GSEA plot of some notable up-regulated and down-regulated enriched gene sets in tumors receiving triple *in situ* vaccine.** (A) Up-regulated enriched gene sets associated with immune activation. (B, C) Down-regulated enriched gene sets associated with (B) amino acids metabolism and (C) DNA repair.

**
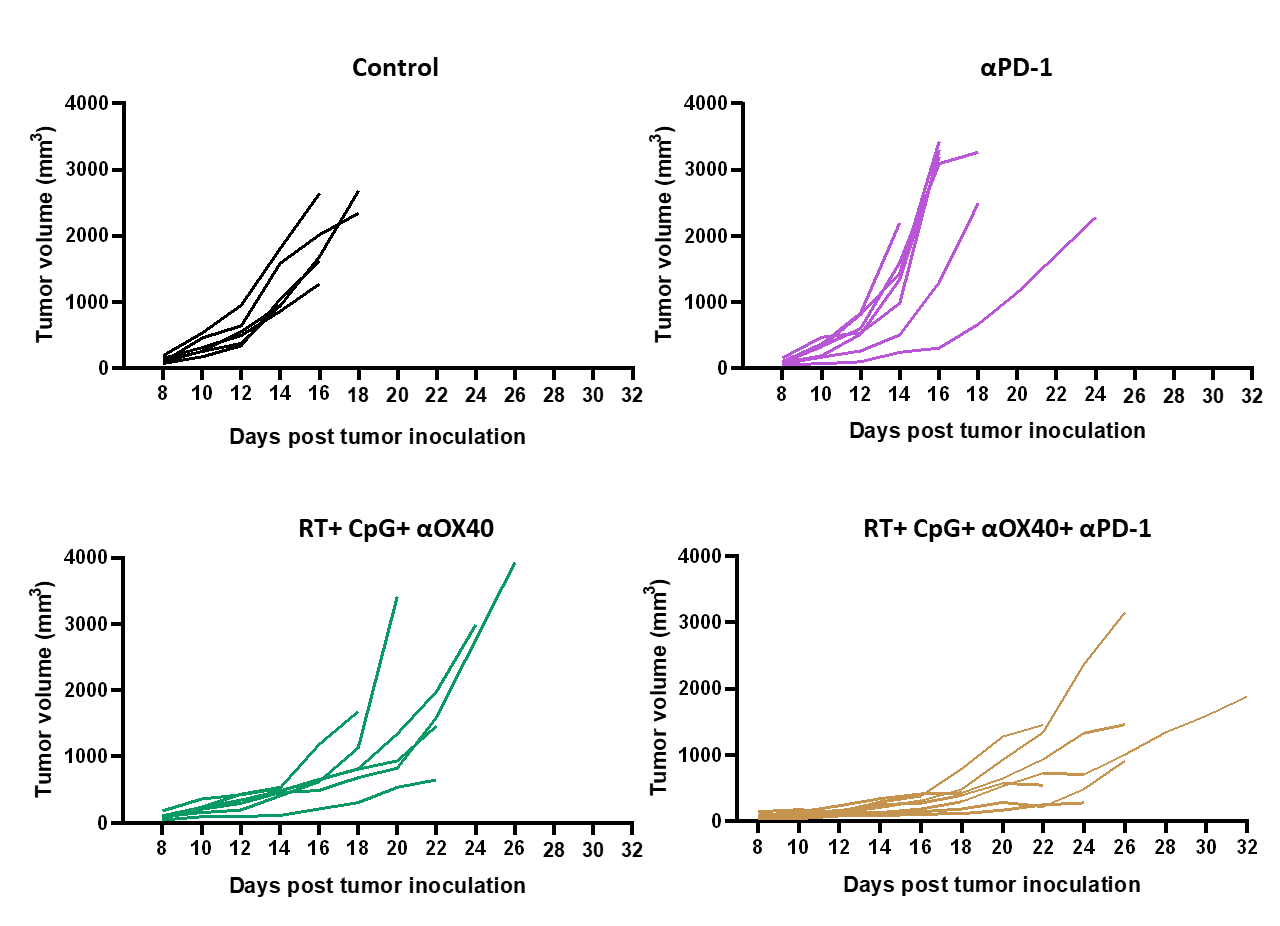
**

**Figure S7.** **Individual tumor growth curves of each mouse in different groups (n = 6-7).**


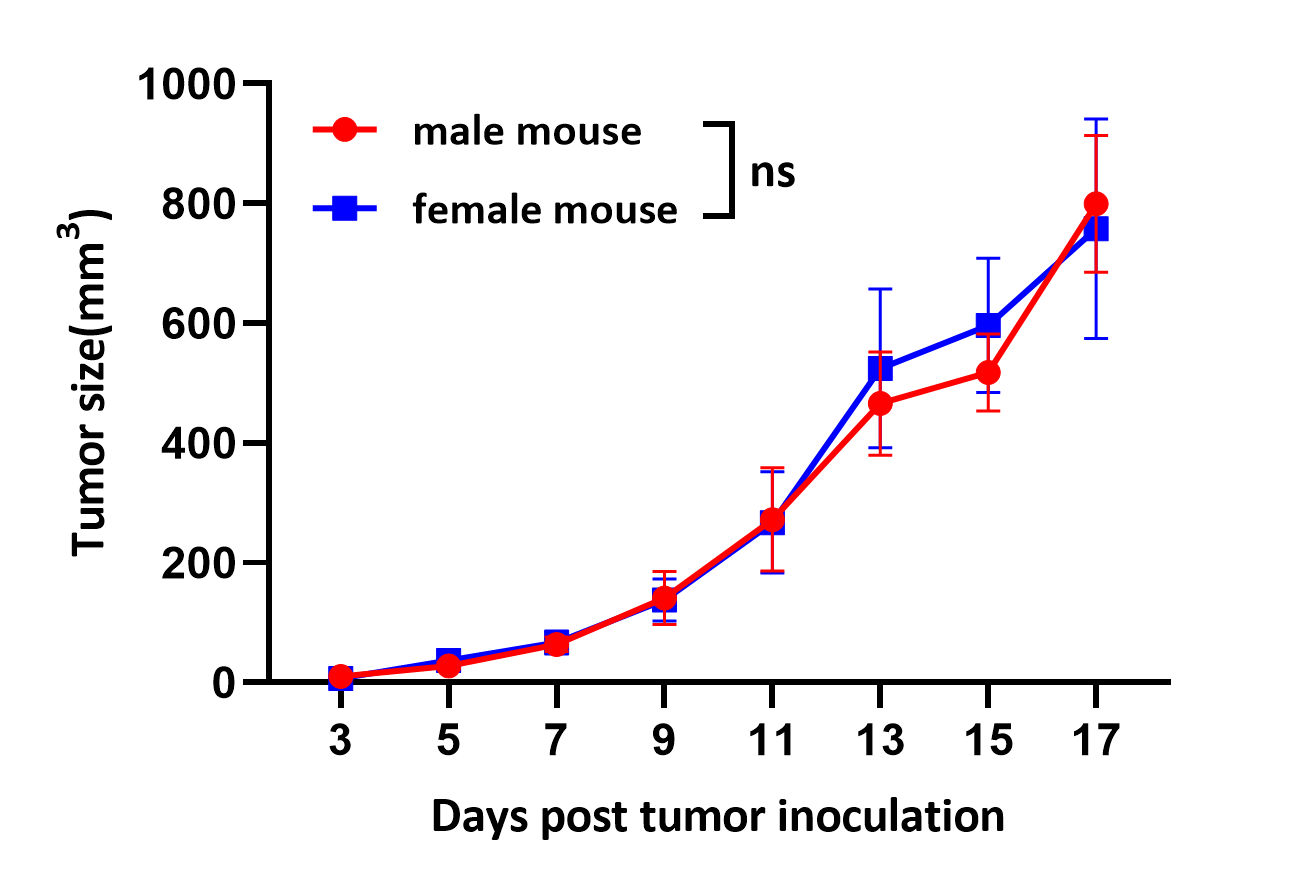


**Figure S8. Tumor growth curves of different genders.** Male and female C57BL/6 mice aged 6-7 weeks, kept under the same condition, were subcutaneously inoculated with 5×10^5^ B16F10 cells. Tumor growth curves were recorded as mentioned in “Methods”. Data are represented as mean ± s.e.m., n=5. Student’s t test was used for statistical analysis. ns, not significant representing *p* > 0.05.
